# TRPM7 modulates endothelial cell functions and VEGFA-associated signaling in diabetic foot

**DOI:** 10.1371/journal.pone.0359834

**Published:** 2026-10-28

**Authors:** Yifeng Cheng, Ling Jia, Li Sheng, Xu Han, Yang Zhao

**Affiliations:** 1 Laboratory of Pathogenesis, Prevention and Treatment of High Incidence Diseases in Central Asia, Urumqi, Xinjiang, China; 2 Department of Burns, The First Affiliated Hospital of Xinjiang Medical University, Urumqi, Xinjiang, China; 3 Medical Plastic Surgery and Cosmetic Center, The First Affiliated Hospital of Xinjiang Medical University, Urumqi, Xinjiang, China; 4 Department of Anesthesiology, The First Affiliated Hospital of Xinjiang Medical University, Urumqi, Xinjiang, China; Advanced Materials Technology Research Institute, National Research Centre, EGYPT

## Abstract

**Background:**

Endothelial dysfunction is a central contributor to the development of diabetic foot complications. However, the regulatory mechanisms underlying impaired vascular endothelial cell (VEC) function in diabetic foot remain incompletely understood. Transient Receptor Potential Melastatin 7 (TRPM7) is implicated in cardiovascular pathology, yet its role in diabetic foot–related endothelial impairment is unclear.

**Methods:**

Primary VECs were isolated from foot vascular tissues obtained from control participants and patients with DFUs and identified by CD31 and factor VIII immunofluorescence. Proliferation, apoptosis, migration, and in vitro tube formation were quantitatively assessed. TRPM7 was overexpressed in control-derived VECs and knocked down using shRNA in DFU-derived VECs. TRPM7 expression and VEGFA–AKT–ERK-related proteins were evaluated using RT-qPCR and Western blotting. Advanced glycation end products (AGEs) and inducible nitric oxide synthase (iNOS) concentrations in conditioned culture supernatants were measured by ELISA.

**Results:**

Compared with control-derived VECs, DFU-derived VECs exhibited reduced proliferation, migration, and tube formation, together with increased apoptosis and elevated conditioned-supernatant AGE and iNOS protein concentrations. TRPM7 expression was higher in DFU-derived VECs. TRPM7 overexpression in control-derived VECs induced a dysfunctional cellular phenotype, whereas TRPM7 knockdown in DFU-derived VECs partially restored proliferation, migration, and tube formation and reduced apoptosis. TRPM7 manipulation was also accompanied by changes in VEGFA expression and AKT and ERK phosphorylation, indicating an association between TRPM7 status and dysregulated VEGFA–AKT–ERK-related signaling.

**Conclusion:**

In primary human VEC cultures, increased TRPM7 expression was associated with impaired endothelial proliferation, migration, and tube formation and with altered VEGFA-related AKT/ERK signaling. This supporting TRPM7 as a candidate regulator of endothelial dysfunction under diabetic-foot–related conditions.

## Introduction

Diabetic foot complications, as one of the common and serious complications of diabetes, have long been a subject of considerable attention [1]. The pathological process involved is highly intricate, with the diminished biological functions of vascular endothelial cells considered a pivotal factor in the development of diabetic foot complications [2]. Endothelial cells, as crucial components of the vascular wall, play vital roles in maintaining vascular function, regulating blood flow, and inhibiting thrombus formation [3]. However, persistent DFU healing failure is associated with high blood glucose levels induces metabolic disturbances in endothelial cells, leading to increased intracellular generation of reactive oxygen species, excessive release of inflammatory factors, and a cascade of cellular stress responses triggered by intracellular glycation damage [4]. Therefore, the therapeutic effects may be heterogeneous or transient and can be influenced by cell source, administered cell number, culture conditions, inflammatory activation, and the local wound microenvironment [5].

Transient Receptor Potential (TRP) channels are a class of non-selective cation channels located on the cell membrane, aiding cells in sensing various stimuli from the external fluid environment [6]. Currently, the TRP family comprises 7 subtypes, including TRPA, TRPC, TRPM, TRPML, TRPN, TRPP, and TRPV [7]. Among them, the TRPM family is exclusive to mammals and plays a crucial role in various physiological and pathological processes such as tumor development, renal diseases, cardiovascular health, and neuronal development [8]. TRPM7, as a member of the TRPM family, is expressed in various tissues and organs throughout the body, participating in numerous physiological and pathological processes, including the transport and balance of ions like Ca^2+^ and Mg^2+^, embryonic development, the occurrence of cardiovascular and neurological diseases, the progression of diabetes, induction of cell apoptosis, and the development of tumors [9].

Recent evidence indicates that diabetes-associated endothelial dysfunction is driven partly by hyperglycemia-induced oxidative stress and disruption of the balance between PI3K-AKT and MAPK-ERK signaling [10]. TRPM7 was prioritized because recent studies have identified it as a context-dependent regulator of endothelial proliferation and vascular homeostasis, while TRPM7 inhibition or knockdown can also alter endothelial angiogenesis by reducing VEGFR2 stability through a Zn^2+^-mTOR-receptor tyrosine kinase mechanism [11,12]. We therefore hypothesized that the diabetic-foot microenvironment dysregulates TRPM7-dependent ionic and metabolic signaling, thereby perturbing the VEGFA–AKT/ERK network and impairing endothelial repair; however, the direction and causality of this relationship require further mechanistic investigation. Therefore, this study aims to investigate the impact of TRPM7 on cell proliferation, vascular formation and migration in diabetic foot by isolating primary human foot vascular endothelial cells and modulating the expression of TRPM7 through knockdown and overexpression approaches.

## Materials and methods

### Cells were separated and cultured

Normal foot tissues from healthy people and diabetic foot tissues from diabetic patients were obtained from the First Affiliated Hospital of Xinjiang Medical University. Participant recruitment was conducted from June 1, 2023 to March 31, 2024. Tissue samples were collected exclusively from patients undergoing clinically indicated surgical procedures, and no additional interventions were performed for research purposes. This study was conducted in accordance with the Declaration of Helsinki and was approved by the Ethics Committee of the First Affiliated Hospital of Xinjiang Medical University (Approval No. K202305-06). All participants provided written informed consent prior to enrollment and tissue collection. Before consent was obtained, each participant received a detailed explanation of the study objectives, procedures, and potential risks. The inclusion criteria for diabetic foot patients were: 1) clinically diagnosed with Type 2 Diabetes Mellitus; 2) presence of diabetic foot ulcers requiring surgical debridement or amputation. The exclusion criteria included: 1) severe cardiovascular or cerebrovascular acute events within the past 6 months; 2) systemic autoimmune diseases; 3) malignant tumors; and 4) acute systemic infections or sepsis. The healthy control tissues were obtained from non-diabetic individuals undergoing trauma-related orthopedic surgeries, with no history of metabolic or peripheral vascular diseases. The matching criteria and comparisons of baseline characteristics are now reported in S1 Table.

The tissues were placed in a laminar flow hood for isolation. Using ophthalmic scissors and forceps, efforts were made to remove fat and fibrous tissue from the outer membrane of blood vessels. Subsequently, the blood vessels were washed several times with PBS containing antibiotics, both on the outer surface and inside the vascular lumen, and then placed in another sterile culture dish. The blood vessels were longitudinally cut open with ophthalmic scissors, spread flat in the culture dish, and cut into tissue pieces of approximately 1 mm × 1 mm in size. These tissue pieces were then treated with 0.1% trypsin (containing antibiotics) at 37°C for 5 min. After gentle agitation and standing for 1 minute, the upper liquid was aspirated. The remaining trypsin activity was neutralized by adding 10 times the volume of 15% FBS culture medium. The mixture was centrifuged at 1000 rpm for 10 minutes at 4°C, and the collected cells were resuspended in 3–5 ml of endothelial cell complete culture medium comprising DMEM, supplemented with 10% fetal bovine serum (FBS, Gibco). The suspension was placed in a 37°C, 5% CO_2_ incubator for 4–6 h. After slow inversion of the culture bottle to ensure the adherent cells were fully immersed in the medium, the cells were left undisturbed for 4–5 days. The medium was changed every 3 days until some cells migrated out from around the tissue blocks. The medium was then replaced, and this process continued until passage. When the cells reached 90% confluence, they were passaged using 0.25% trypsin and used for subsequent experiments.

### Immunofluorescent staining

After washing the processed cells with PBS once, they were fixed with 4% paraformaldehyde for 15–30 min. Subsequently, they were treated with 0.1% Triton X-100 at room temperature for 15 min. This step is for cell permeabilization, and the duration may vary based on the location of the target protein expression. Antibodies targeting membrane expression do not require permeabilization, while those targeting cytoplasmic and nuclear expression necessitate this step. After permeabilization, the cells were washed twice with PBS and then blocked with 5% FBS at room temperature for 15 min. Primary antibodies against CD31 (1:500, Abcam, CAT: ab182981) and Factor VIII (1:1000, Abcam, CAT: ab275376) were applied and left to incubate overnight at 4℃. The cells were then washed three times with PBS, each time for 5 min. Subsequently, secondary antibodies (1:200, Proteintech, CAT: SA00009−2) were added, and the cells were incubated at 37℃ for 1 hour. Afterward, the cells were incubated with Hoechst at room temperature in the dark for 15 min. Finally, fluorescent images were captured using a fluorescence microscope.

### Fluorogenic quantitative PCR (RT-PCR)

Total RNA was extracted from VECs using TRIzol reagent (Invitrogen, CAT: 15596018). Synthesize cDNA using the first-strand cDNA synthesis kit (Thermo, CAT: K1622). After PCR amplification, the results were automatically analyzed by a real-time fluorescence quantitative PCR instrument (Thermo, CAT: F-415XL). Primer sequences were: TRPM7: Forward, CTCTTCTGGTGCCTTATTCCC; Reverse, GCAACTTGGCTGAGATGGTGT. GAPDH: Forward, GGAGCGAGATCCCTCCAAAAT; Reverse, GGCTGTTGTCATACTTCTCATGG. Adjust the threshold and baseline according to the negative control to determine the Ct value of each sample. Analyze the expression differences of the target gene between the control group and each experimental group using the 2^-ΔΔCT^ method.

### Western Blotting

To lyse the treated cells and assess protein concentration, 5 × SDS was utilized, followed by boiling in a water bath at 95℃ for 10 min. For SDS-PAGE gel preparation, a 12% separating gel and a 5% stacking gel were configured, and protein samples were subjected to electrophoresis. Subsequent to electrophoresis, transfer, and blocking, primary antibody immunoblotting (TRPM7, 1:1000, Abcam, Cat: ab245408; VEGFA, 1:1000, Abcam, Cat: ab46154; p-AKT, 1:1000, Abcam, Cat: ab81283; AKT, 1:1000, Abcam, Cat: ab8805; p-ERK, 1:1000, Proteintech, Cat: 80031–1-RR; ERK, 1:1000, Proteintech, Cat: 66192–1-Ig; and β-actin, 1:2000, Abcam, Cat: ab8227) was performed overnight on a shaker at 4℃. Membranes were washed three times with 1 × TBST, each time for 10 min. Subsequently, membranes were incubated with the corresponding secondary antibody (1:2000, Proteintech, Cat: RGAR001) at room temperature for 1 h. After washing the membranes three times with 1 × TBST for 10 min each time, ECL luminescent solution was applied for color development, followed by image capture. Band intensities were quantified using ImageJ. TRPM7, VEGFA, AKT, and ERK expression was normalized to β-actin, whereas AKT and ERK phosphorylation was expressed as the p-AKT/total AKT and p-ERK/total ERK ratios, respectively. Protein bands were analyzed using Image J.

### Enzyme linked immunosorbent assay (Elisa) of AGEs and iNOS

The concentrations of AGEs and iNOS-immunoreactive protein in the culture supernatants were determined using commercial ELISA kits (AGEs, Mibio, Cat: ml105237; iNOS, Mibio, Cat: ml061044) according to the manufacturer’s instructions. Remove the required slats from the aluminum foil bag after 10 min of room temperature equilibrium, and the remaining slats are sealed in a ziplock bag and returned to 4℃. Adding sample: Add the sample or standard product of different concentration into the corresponding hole according to 50 μl per hole, add 50 μL general diluent to the blank hole, and then add 50 μL HRP-antigen working liquid to each hole. Cover the sealing film and incubate at 37℃ for 1 h. Discard the liquid, add 300 μL 1x washing liquid to each well, leave for 1 minute, shake off the washing liquid, pat dry on absorbent paper, repeat washing plate 5 times. Add substrate (TMB) 90 μL to each hole, cover the sealing plate film, warm at 37℃ for 15 min, add termination solution 50 μL to each hole, and immediately determine the OD value of each hole at 450 nm wavelength.

### Cell transfection

The human TRPM7 overexpression construct was generated by Shanghai GeneChem Co., Ltd. using the GV747 lentiviral expression vector. The vector contained a CMV enhancer, a multiple-cloning site, and a T2A-puromycin cassette. The complete human TRPM7 coding sequence corresponding to NCBI RefSeq NM_017672 was inserted into the BamHI and XhoI cloning sites. The amplified TRPM7 insert was 5,642 bp in length. The primers used for cloning were: Forward primer, TRPM7-p1: 5′-AGGTCGACTCTAGAGGATCCCGCCACCATGTCCCAGAAATCCTGGATAGAAAGC-3′; Reverse primer, TRPM7-p2: 5′-CCCTCGCCGCTGCCCTCGAGTAACATCAGACGAACAGAATTAGTTGATTC-3′. The resulting construct was designated OE-TRPM7, and the inserted sequence was verified by sequencing. VECs derived from non-diabetic control donors were transduced with OE-TRPM7 at a multiplicity of infection of MOI = 100. Cells were incubated with the lentiviral particles for 24 h. The medium was subsequently replaced with fresh complete endothelial-cell medium, and the cells were cultured for an additional 48 h. TRPM7 overexpression was verified using RT-qPCR before functional experiments. The overexpression negative-control group (OE-NC) was transduced with an empty GV747 lentiviral vector lacking the TRPM7 coding sequence.

TRPM7 knockdown was performed using the LV3(H1/GFP&Puro)-TRPM7-homo-2245 shRNA plasmid supplied by GenePharma. The plasmid contained an H1 promoter-driven shRNA cassette, GFP, and a puromycin-selection element. The TRPM7 target sequence was: 5′-GCCTGTAAGATCTATCGTT-3′. The shRNA oligonucleotide sequences were: Sense: 5′-GATCCGCCTGTAAGATCTATCGTTTCAAGAGAACGATAGATCTTACAGGCTTTTTTG-3′; Antisense: 5′-AATTCAAAAAAGCCTGTAAGATCTATCGTTCTCTTGAAACGATAGATCTTACAGGCG-3′. VECs derived from patients with diabetic foot were seeded in six-well plates and cultured until they reached approximately 80% confluence. For each well, 10 μg of the TRPM7 shRNA plasmid was mixed with 4 μL Lipo8000 transfection reagent in serum- and antibiotic-free medium according to the manufacturer’s protocol. After incubation for 30 min at room temperature, the transfection mixture was added to the cells. After 6 h, the medium was replaced with complete endothelial-cell medium, and the cells were cultured for another 48 h. TRPM7 knockdown efficiency was verified by RT-qPCR before subsequent assays. The knockdown negative-control group (shRNA-NC) was transfected with a non-targeting shRNA plasmid containing the same LV3(H1/GFP&Puro) backbone.

### CCK8

After the cells reached confluence, the cell concentration was adjusted to 1 × 10^4^ cells/mL. Subsequently, they were seeded into a 96-well culture plate, with each well containing 100 μL. According to the experimental groups mentioned above, the corresponding viruses were inoculated. The cells were cultured for 24 h, 48 h, and 72 h, and then 20 μl of CCK-8 (Beyotime, Cat: C0037) was added to each well. The plates were further incubated at 37℃ in a 5% CO_2_ incubator without light for 4 h. The absorbance at a wavelength of 450 nm was measured using a microplate reader to analyze the impact on cell growth based on the obtained OD values at the same time points.

### Cell apoptosis

Cell apoptosis was evaluated using an Annexin V-FITC/PI apoptosis detection kit (BD, USA, Cat: 556574) according to the manufacturer’s instructions. After the indicated treatments, both floating and adherent cells were collected. Adherent cells were detached using trypsin without EDTA and combined with the corresponding culture supernatants. The cells were centrifuged at 1,000 rpm/300 × g for 5 min, washed twice with cold PBS, and resuspended in 1 × binding buffer at approximately 1 × 10^6^ cells/mL. A 100-μL aliquot of the cell suspension, containing approximately 1 × 10^5^ cells, was incubated with 5 μL Annexin V-FITC and 5 μL PI for 15 min at room temperature in the dark. Subsequently, 400 μL of 1 × binding buffer was added, and the samples were analyzed within 1 h using a flow cytometer (BD FACSCelesta). Data were analyzed using FlowJo. The total apoptotic rate was calculated as the sum of the percentages of early apoptotic cells (Annexin V-FITC ⁺ /PI^-^) and late apoptotic cells (Annexin V-FITC ⁺ /PI⁺).

### Cell tubule formation assay

Matrigel matrix gel (Aladdin, B753082-GP) was thawed overnight at 4 ℃, and pipette tips, EP tubes, and 24-well plates were pre-cooled overnight at 4 ℃. The next day, gel overlay was performed on ice by adding 250 μl of Matrigel matrix gel to each well of a 24-well plate, followed by incubation at 37℃ for 30 min. During the gel solidification process, cell suspensions were prepared (cells grouped according to previous specifications). After cell digestion, the cell suspension was adjusted to a concentration of 2 × 10^5^ cells/mL. Then, 50,000 cells were seeded into each well and incubated for 48 h. Observation and imaging were performed under a fluorescence microscope after 48 h.

Take the prepared cell samples, add 3 ml of PBS to wash the cells, digest with 0.25% trypsin, centrifuge at 1000 rpm for 5 min, discard the supernatant, rinse with PBS twice to remove residual serum. Suspend cells in serum-free DMEM medium, count the cells using a cell counting plate, dilute the cell concentration to 3 × 10^5^ cells/ml in serum-free 1640 medium, and set aside. Pre-add 800 μl of 10% FBS-containing 1640 medium to a 24-well plate and place it in the transwell chamber. After 1 h, add 200 μl of each group of cell suspensions to the upper chamber of the transwell, and incubate at 37 °C in a 5% CO_2_ incubator for 48 hours. Remove the transwell, carefully wash the chamber with PBS, fix the cells with 70% ice-cold ethanol for 1 hour. Stain with 0.5% crystal violet staining solution, place at room temperature for 20 min, rinse with PBS, use a clean cotton ball to wipe off the unmigrated cells on one side of the upper chamber, and observe and photograph under a microscope.

### Statistical analysis

Statistical analyses were performed using GraphPad Prism 10 (GraphPad Software, MA, USA). Data are presented as the mean ± SD of n = 6 independent donor-derived VEC preparations per group. Each experiment was independently repeated 3 times. Comparisons between VECs obtained from healthy donors and diabetic-foot donors were performed using a two-sided Welch’s t-test. One-way ANOVA was used for experiments in which cells from the same donor, followed by Dunnett’s multiple-comparisons test. P < 0.05 was considered statistically significant.

## Results

### Isolation and culture of VECs in human foot

In order to investigate the changes in VECs during the diabetic foot process, we utilized enzymatic digestion to extract VECs from the foot tissues of both healthy individuals and diabetic patients. Primary cells isolated from the foot vascular tissues adhered to the culture surface and gradually proliferated around the tissue fragments. At confluence, the cells exhibited a polygonal, cobblestone-like morphology under microscopy (Fig 1A). Immunofluorescence staining demonstrated CD31 and Factor VIII-related antigen immunoreactivity in the isolated cell cultures (Fig 1B, C), supporting an endothelial phenotype. These findings suggest the successful isolation of VECs from the feet of both normal individuals and diabetic patients.

**Fig 1:**
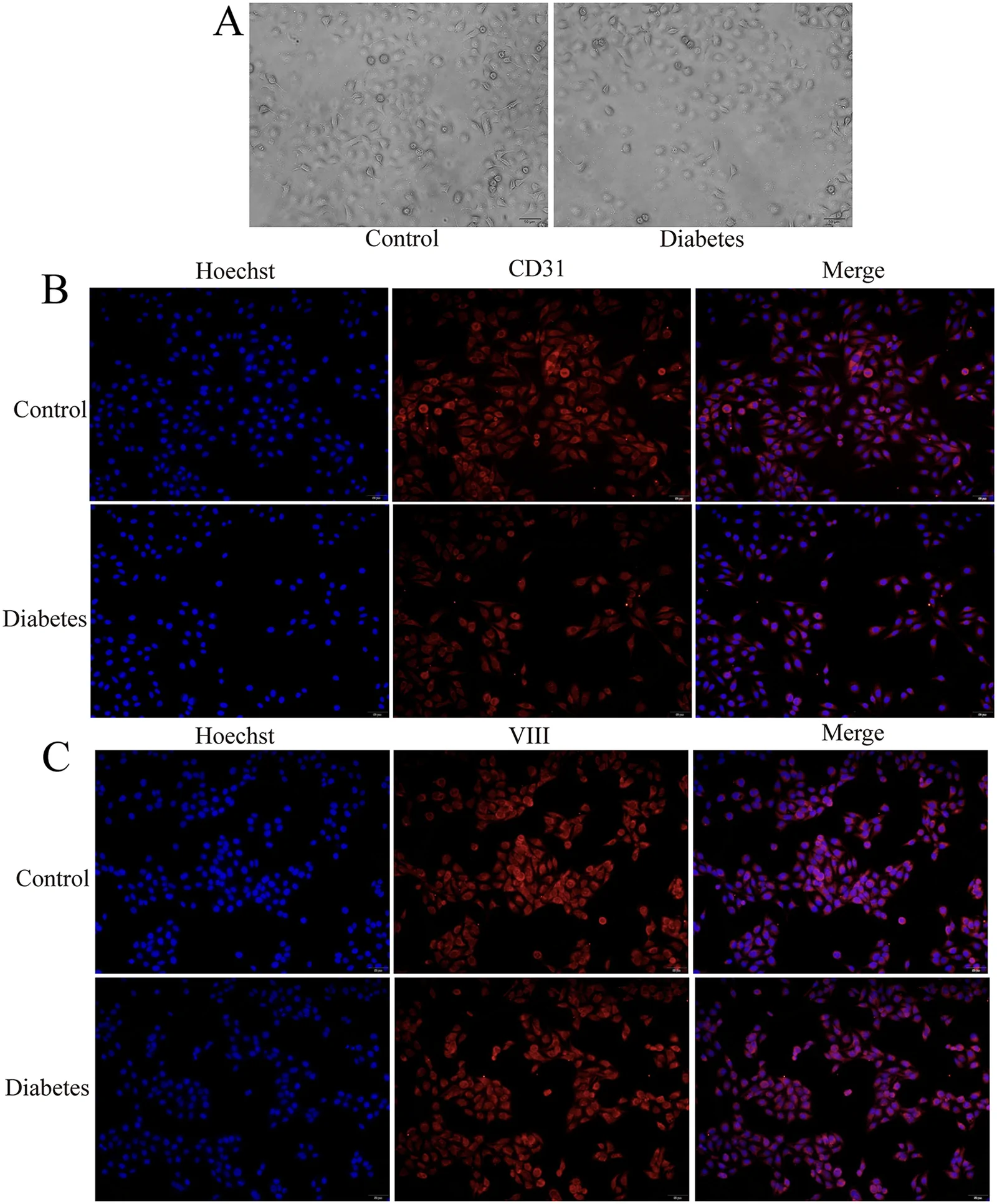
Characterization of primary vascular endothelial cells isolated from foot tissues of healthy individuals and patients with diabetic foot. (A) Representative images showing the morphology of primary cells derived from non-diabetic control donors and patients with diabetic foot. (B) Representative immunofluorescence images showing CD31 staining. (C) Representative immunofluorescence images showing Factor VIII-related antigen staining. Nuclei were counterstained with Hoechst.

### Changes in biological function of VECs during diabetic foot

To investigate the effect of diabetes on the function of human primary vascular endothelial cells, we subsequently conducted CCK8 assays, apoptosis, tube formation experiments, and transwell assays on VECs to assess the effects of diabetes. The results revealed that diabetes significantly reduced the cell proliferation (Fig 2A), and increased the levels of apoptosis (Fig 2B) in VECs. Additionally, diabetes markedly decreased the tube formation ability of vascular endothelial cells (Fig 2C, D). Furthermore, diabetes significantly inhibited the migration ability of vascular endothelial cells (Fig 2E, F). Conditioned media collected from diabetic foot-derived VECs contained significantly higher concentrations of AGEs and iNOS-immunoreactive protein than media collected from control VECs (Fig 2G and H). These findings indicate that diabetes significantly impairs the biological functions of vascular endothelial cells.

**Fig 2:**
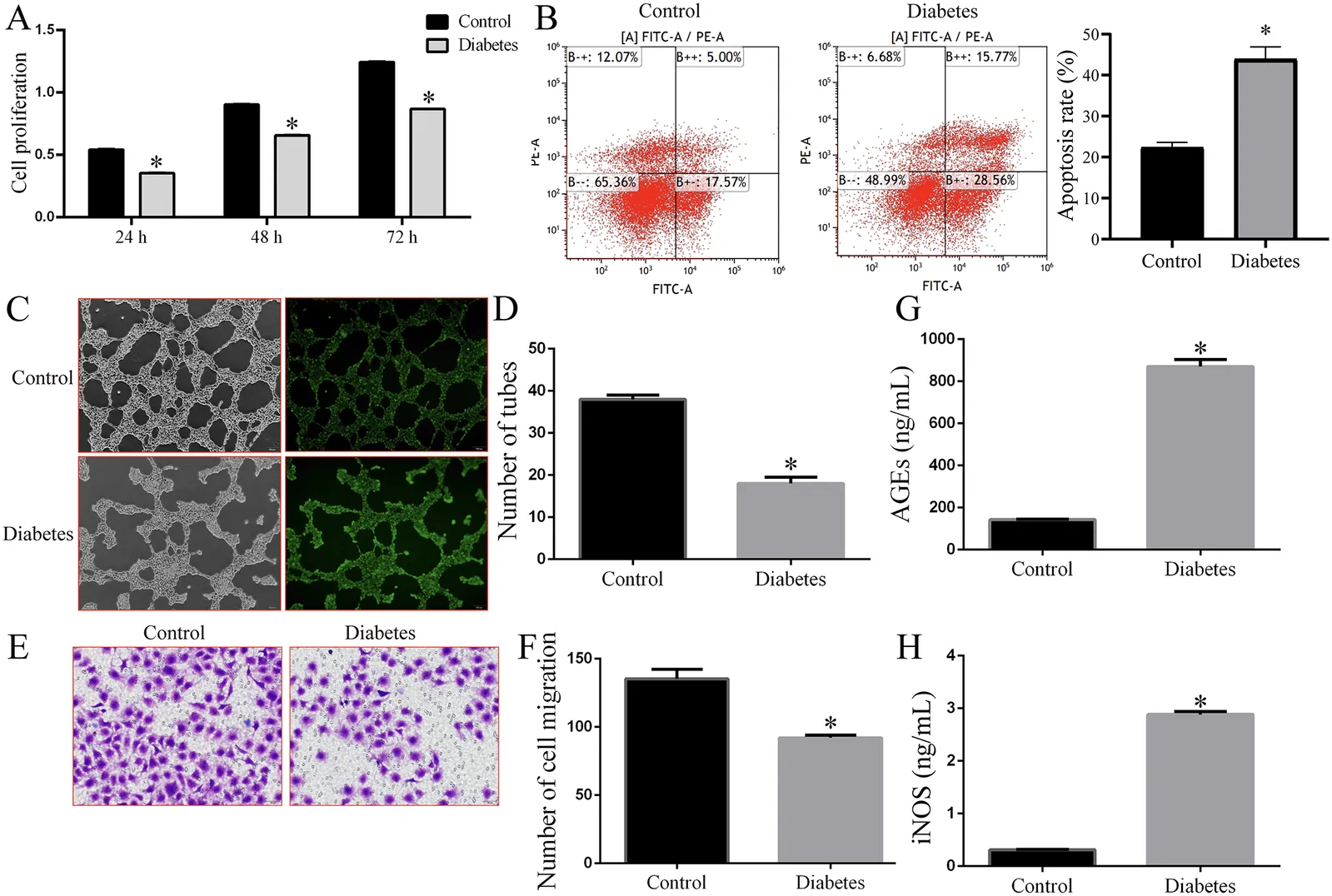
Changes in biological function of VECs during diabetic foot. (A) The VECs were seeded in a 96-well plate. Cell proliferation was assessed using CCK8 at 24 h, 48 h, and 72 h. (B) Apoptosis detection kit was used to detect cell apoptosis. The apoptotic rate was expressed as the percentage of apoptotic cells among the total analyzed cells. (C, D) The VECs were plated on Matrigel-coated 24-well plates, and tube formation ability was observed after 48 h. (E, F) Transwell assay was employed to assess the migration ability in VECs. (G) The levels of AGEs and iNOS were detected by Elisa. p <  0.05, * indicates comparison with the Control group, p <  0.05.

### Expression of TRPM7 in VECs from the feet of healthy individuals and diabetic patients

The elevated expression of TRPM7 has been shown in previous studies to be significantly associated with an increased incidence of cardiovascular diseases. Therefore, we speculated that the inhibitory effect of diabetes on the biological functions of vascular endothelial cells (VECs) might depend on the regulatory role of TRPM7. To verify this hypothesis, we detected the mRNA expression levels of TRPM7 in VECs from the feet of both healthy individuals and diabetic patients. The results revealed a significant increase in the mRNA expression levels of TRPM7 in VECs from diabetes foot (Fig 3). These findings suggest that TRPM7 may play a crucial role in mediating the inhibitory effects of diabetes on the biological functions of VECs.

**Fig 3:**
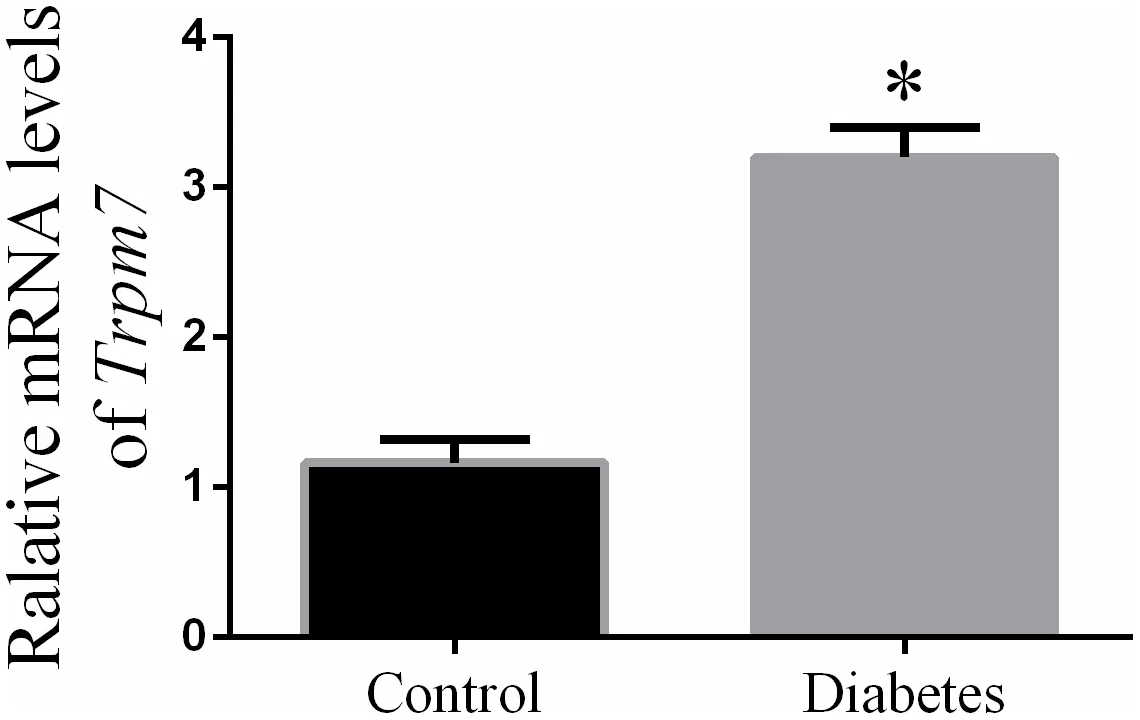
The expression levels of TRPM7 in VECs from diabetes foot. * indicates comparison with the Control group, p <  0.05.

### The role of TRPM7 in suppressing VECs biological functions

To investigate the role of TRPM7 in attenuating the biological functions of vascular endothelial cells in diabetes. Firstly, we constructed overexpression and knockdown shRNA of TRPM7, and detected the mRNA expression levels of TRPM7. The results showed that we successfully knocked down or overexpressed TRPM7 in VECs (Fig 4A). Subsequently, TRPM7 was overexpressed in VECs from healthy individuals foot and knocked down TRPM7 in VECs from diabetic foot. We then assessed changes cell proliferation (Fig 4B), apoptosis (Fig 4C), tube formation ability (Fig 4D and Fig 4E), and migration ability (Fig 4F and Fig 4G) in VECs. The results indicated that overexpression of TRPM7 from healthy individuals significantly inhibited cell proliferation, tube formation, and migration abilities in VECs. Meanwhile, overexpression of TRPM7 significantly increased the expression levels of AGEs and iNOS in VECs (Fig 4H and Fig 4I). Conversely, knocking down TRPM7 from diabetic patients promoted cell proliferation, tube formation ability, and migration ability in VECs, and reduced apoptosis. Moreover, knocking down TRPM7 reduced the levels of AGEs and iNOS (Fig 4H and Fig 4I). These findings suggest that diabetes affects the biological functions through TRPM7 in VECs.

**Fig 4:**
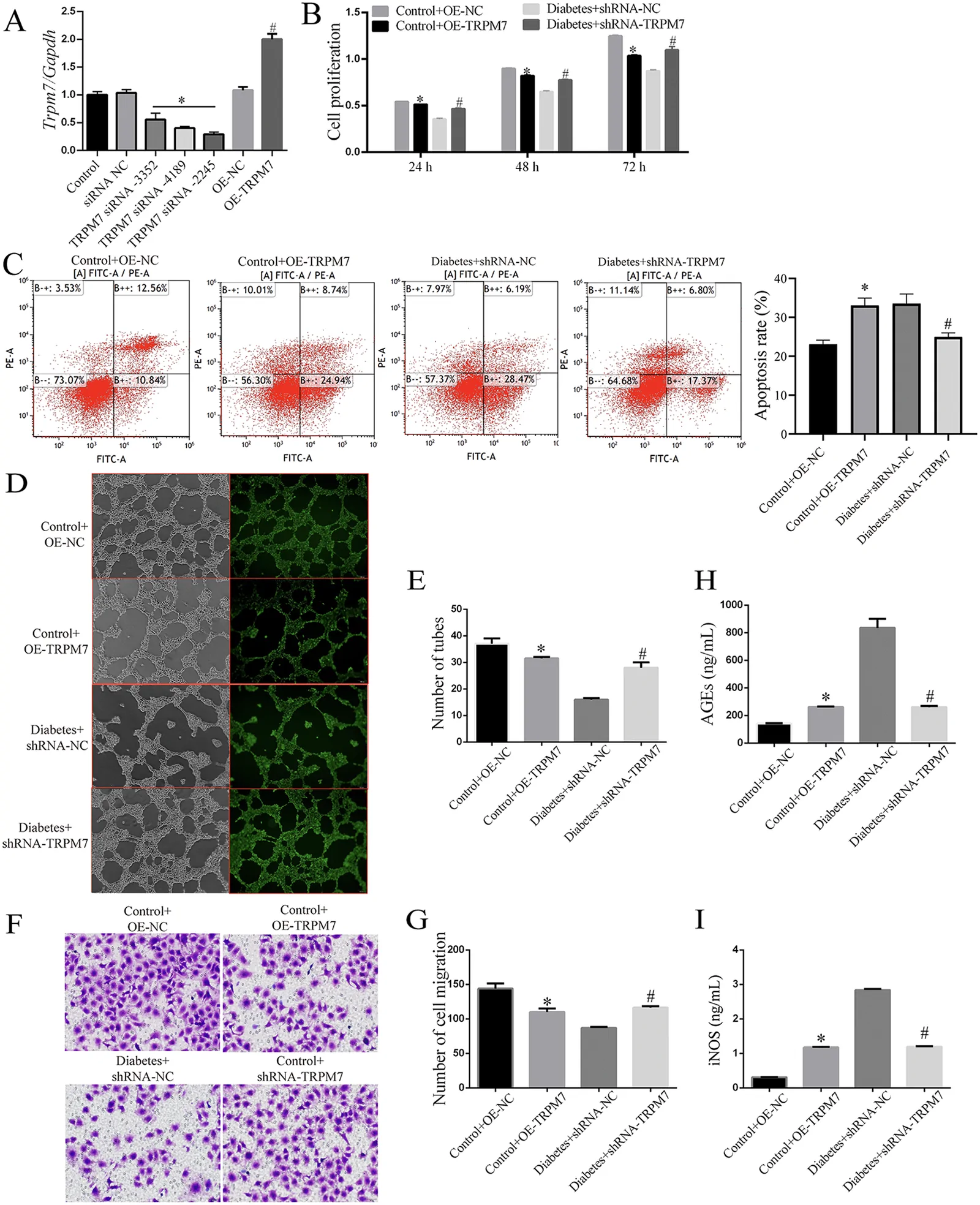
Effects of TRPM7 overexpression and knockdown on VEC functions. (A) TRPM7 was overexpressed in VECs derived from non-diabetic control donors by transduction with packaged LV-TRPM7 lentiviral particles. Control + OE-NC cells were transduced with matched empty GV747 lentiviral particles. TRPM7 was knocked down in diabetic-foot VECs by Lipo8000-mediated transfection of the LV3(H1/GFP&Puro)-TRPM7-homo-2245 shRNA plasmid. Diabetes + shRNA-NC cells were transfected with a matched non-targeting shRNA plasmid containing the same LV3 backbone. Subsequently, changes in (B) cell proliferation, cell apoptosis (C), tube formation ability (D, E) and migration capability (F, G) of VECs were detected. (H. I) The levels of AGEs and iNOS were detected by Elisa. *indicates comparison with the Control+OE-NC group, #indicates comparison with the Diabetes+shRNA-NC, *p* <  0.05.

### TRPM7 manipulation is associated with altered VEGFA–AKT–ERK signaling markers

To further investigate the impact of TRPM7 on the biological functions of VECs, we subsequently examined the status of the VEGFA-AKT-ERK signaling pathway in VECs from both the healthy control group and diabetic foot patients (Fig 5 and S1 Fig). The results indicate a significant increase in the protein expression levels of VEGFA, p-ERK, and p-AKT in VECs from diabetic foot patients. These molecular changes occurred together with reduced proliferation, migration, and tube formation and increased apoptosis.

**Fig 5:**
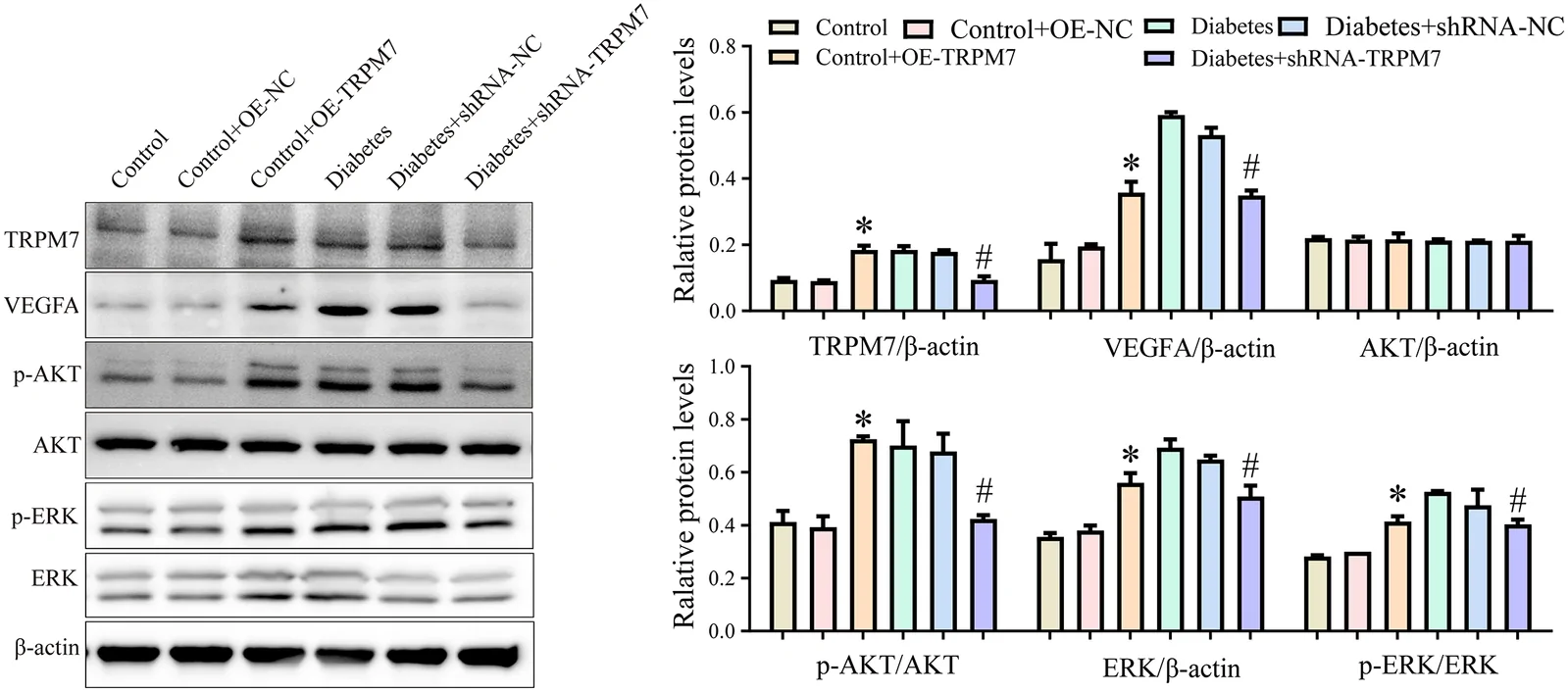
TRPM7 is associated with activation of the VEGFA-AKT-ERK signaling pathway in Diabetes foot. TRPM7 was knocked down or overexpressed in VECs from the feet of healthy individuals and diabetic foot patients. Protein expression levels of TRPM7, VEGFA, ERK, p-ERK, AKT, and p-AKT were assessed using the Western blot method. Original blots are presented in S1 Fig. * indicates significant compared to Control+OE-NC group, # indicates significant compared to Diabetes+shRNA-NC group, *p* <  0.05.

Subsequently, we overexpressed TRPM7 in VECs from the feet of healthy individuals and knocked down TRPM7 in VECs from the feet of diabetic foot patients. The status of the VEGFA-AKT-ERK signaling pathway was then assessed. The results indicate that overexpression of TRPM7 in VECs from healthy individuals’ feet significantly increased the protein expression levels of TRPM7, VEGFA, p-ERK, and p-AKT. These findings suggest that TRPM7 can significantly altered the VEGFA-AKT-ERK signaling pathway. Additionally, knocking down TRPM7 in VECs from diabetic foot patients’ feet significantly decreased the protein expression levels of TRPM7, VEGFA, p-ERK, and p-AKT.

## Discussion

In the present study, primary VECs isolated from diabetic-foot tissues exhibited reduced proliferation, migration, and tube formation, together with increased apoptosis. TRPM7 expression was elevated in diabetic-foot VECs, and genetic manipulation of TRPM7 altered these cellular phenotypes under in vitro conditions. TRPM7 overexpression and knockdown were also accompanied by corresponding changes in VEGFA expression and AKT/ERK phosphorylation. Collectively, these findings suggest that TRPM7 may participate in the regulation of endothelial dysfunction and is associated with dysregulated VEGFA-related AKT/ERK signaling. Nevertheless, the present results do not demonstrate that TRPM7 is an indispensable mediator of diabetic-foot pathology in vivo or that the observed cellular effects are mediated exclusively through this signaling pathway.

Diabetic foot pathology is a severe complication among individuals with diabetes, characterized by a complex interplay of factors involving the nervous, vascular, immune, and inflammatory systems [13]. Notably, VECs play a crucial yet intricate role in the onset of diabetic foot. Prolonged hyperglycemia in diabetic patients leads to dysfunctional VECs, affecting their proliferation, migration, apoptosis, and the production of inflammatory factors [14]. These aberrant changes can disrupt vascular structure and function, ultimately triggering the development of diabetic foot complications [15]. Research indicates that elevated blood glucose induces oxidative stress in endothelial cells, increases the release of inflammatory factors, and contributes to apoptosis and endothelial membrane damage [16]. The diminished biological functions of endothelial cells emerge as a pivotal factor in the pathogenesis of diabetic foot complications [17].

In this study, the proliferation, tube-forming ability, and migratory capacity of diabetic foot VECs were notably inhibited, accompanied by a significant increase in apoptosis levels. The compromised biological functions of endothelial cells contribute to the disruption of vascular structure and function, playing a key role in the development of diabetic foot complications. Investigating methods to alleviate diabetes-induced suppression of endothelial cell biological functions may provide a theoretical foundation for the future treatment of diabetic cardiovascular diseases. The effects of TRPM7 manipulation on endothelial proliferation, apoptosis, migration, and tube formation may involve both relatively proximal TRPM7-dependent events and secondary downstream responses. As a bifunctional channel-kinase, TRPM7 can potentially initiate cellular responses through divalent-cation transport and kinase-dependent phosphorylation. These early events may subsequently influence receptor stability, cellular metabolism, redox homeostasis, cytoskeletal organization, and transcriptional regulation. In a different vascular context, endothelial TRPM7 deficiency altered glycolytic lactate production and H3K18 lactylation, resulting in p21-dependent endothelial senescence and impaired angiogenesis [18]. These indicate that TRPM7-dependent endothelial phenotypes can be mediated through diverse metabolic and signaling intermediates and may vary according to the disease context and duration of TRPM7 perturbation.

TRPM7 is an essential non-selective cation channel on the cell membrane, playing a crucial role as a voltage sensor and serving as a necessary channel for organism development [19]. The structure of TRPM7 consists of three main regions: the N-terminus, channel domain, and C-terminus [20]. The transmembrane channel structure, located in the transmembrane space, includes the pore loop (P-loop) that forms the ion-conducting pathway [21]. Additionally, the C-terminus of TRPM7 comprises two domains: the serine/threonine protein kinase structure and the α-kinase domain [22]. The TRPM7 channel and kinase domains can interact, and the channel activity is negatively regulated by intracellular cations and Mg-ATP, among other factors [23]. The distinctive structural features of TRPM7 enable it to regulate ion distribution and participate in intracellular signal transduction [24]. Studies have demonstrated that TRPM7 exhibits the highest expression levels in the cardiovascular system and is involved in regulating various physiological and pathological processes, including cell proliferation, vitality, migration, adhesion, and apoptosis [25]. However, the impact of TRPM7 on the biological functions of endothelial cells remains unknown. In this study, we observed a significant upregulation of TRPM7 expression in VECs from diabetic patients. This finding further suggests that TRPM7 may play a crucial role in the inhibition of VECs’ biological functions in diabetes. Subsequently, we manipulated TRPM7 expression in VECs to assess its impact on their biological functions. The results revealed that TRPM7 negatively regulates VECs’ proliferation, tube formation ability, and migration.

The VEGFA-AKT-ERK signaling pathway is a paramount intracellular signaling network participating in the regulation of diverse physiological and pathological processes [26]. VEGFA, a representative member of the VEGF family, acts as a potent inducer promoting angiogenesis [27]. Angiogenesis is a fundamental process in embryonic development, tissue repair, and responding to physiological demands [27]. In the context of angiogenesis, AKT plays a crucial role in promoting endothelial cell survival, preventing programmed cell death, and maintaining the formation and integrity of a functional vascular system [28]. As a member of the mitogen-activated protein kinase family, ERK is crucial for the regulation of cell proliferation [29]. In the context of angiogenesis, the robust proliferation response of endothelial cells is significantly dependent on the activation of ERK, which is essential for the expansion and remodeling of vascular networks [30]. The VEGFA-AKT-ERK signaling pathway is not only indispensable for physiological processes such as embryonic development and tissue repair but is also intricately linked to various pathological conditions [31].

Although our results show that TRPM7 manipulation is accompanied by changes in VEGFA abundance and AKT/ERK phosphorylation, the available evidence supports a context-dependent regulatory association rather than a directly established linear TRPM7-VEGFA-AKT-ERK cascade. Previous endothelial studies provide a biological rationale for this association. In HUVECs, TRPM7 silencing attenuated reactive oxygen species generation and cytotoxicity, prevented the high-glucose-induced reduction in ERK1/2 phosphorylation, and exerted a protective effect that was diminished by the MEK1/2 inhibitor U0126 [25]. Under basal culture conditions, TRPM7 silencing also increased MEK/ERK phosphorylation and endothelial proliferation, and inhibition of ERK signaling abolished the proliferation-promoting effect of TRPM7 knockdown [32]. Zeng et al. further demonstrated that TRPM7 silencing reduced endothelial adhesion but enhanced migration, wound closure, and tube formation, with ERK signaling participating in several of these phenotypic changes [33]. Collectively, these findings indicate that TRPM7 expression and function can influence endothelial oxidative status, ERK signaling, and angiogenic behavior, although the direction of the ERK response may vary with endothelial origin and experimental context.

VEGFA binding to VEGFR2 normally activates PI3K/AKT and ERK signaling branches that promote endothelial survival, proliferation, migration, and angiogenesis [34]. Consistent with these canonical functions, VEGF stimulation has generally shown beneficial effects on diabetic-wound angiogenesis and healing in experimental studies [35]. The increased VEGFA abundance and AKT/ERK phosphorylation may represent an insufficient compensatory or maladaptive response to endothelial injury. Diabetic conditions promote AGE accumulation, oxidative stress, inflammation, and endothelial-cell death. AGE–RAGE signaling can engage PI3K/AKT and Ras-MEK-ERK pathways while simultaneously increasing ROS production, apoptosis, and endothelial dysfunction [10,36]. Consequently, increased phosphorylation of selected signaling proteins may coexist with impaired angiogenic function when glycoxidative and inflammatory stresses override or uncouple the normal VEGFA response.

The AGE and iNOS findings provide additional context for interpreting TRPM7-associated VEGFA-AKT/ERK signaling. AGEs can bind to RAGE and activate multiple oxidative and inflammatory pathways, including NADPH oxidase/ROS, NF-κB, PI3K/AKT, and Ras-MEK-ERK signaling. In diabetic wounds, persistent AGE–RAGE activation has also been associated with impaired endothelial migration, apoptosis, and dysregulated VEGF-dependent vascular repair [10,36]. Thus, the increased AGE levels observed after TRPM7 overexpression may indicate a TRPM7-associated increase in glycoxidative or metabolic stress that converges on the same AKT/ERK signaling network.

Recent experimental evidence indicates that AGE–RAGE signaling can activate Nox1/4-dependent ROS production and impair NO-mediated endothelial vasodilation [37]. AGE-associated inflammatory signaling can also impair endothelial proliferation, migration, and tube formation and increase endothelial adhesion molecules and inflammatory mediators [38,39]. However, direct AGE-dependent regulation of TRPM7 in endothelial cells has not been established. Furthermore, iNOS is commonly induced by inflammatory stimuli and can generate sustained amounts of NO. When superoxide production is increased, NO may react with superoxide to generate peroxynitrite, potentially promoting protein nitration, mitochondrial injury, endothelial barrier impairment, and inflammatory amplification [40]. Thus, the elevated supernatant iNOS concentration observed in diabetic-foot-derived VEC cultures is consistent with an inflammatory or injury-associated endothelial phenotype.

This study has several limitations. First, all functional and molecular experiments were conducted in vitro using primary human VECs. Although these cells retain greater clinical relevance than immortalized endothelial cell lines, the culture system does not reproduce the complex interactions among endothelial cells, inflammatory cells, pericytes, fibroblasts, nerves, and extracellular matrix components within diabetic-foot wounds. Second, the endothelial identity of the isolated primary cells was assessed by qualitative immunofluorescence staining for CD31 and Factor VIII-related antigen, together with supportive evidence from the tube-formation assay. However, no quantitative purity assessment or negative lineage-marker analysis was performed. Therefore, the presence of a small proportion of contaminating non-endothelial cells cannot be completely excluded. Future studies should combine quantitative flow cytometry or immunofluorescence for multiple endothelial markers, such as VE-cadherin/CDH5, VEGFR2/KDR, and ERG, with negative lineage markers such as CD45, α-SMA, and PDGFRβ. An additional limitation is that the present study cannot distinguish direct TRPM7-dependent effects from secondary effects mediated by downstream signaling alterations. The experiments involved prolonged TRPM7 overexpression or knockdown, during which multiple metabolic, transcriptional, and signaling adaptations may have occurred. Functional rescue experiments using TRPM7 channel- or kinase-deficient mutants, VEGFA/VEGFR2 blockade, and AKT or ERK inhibitors will be required to determine the causal contribution of each signaling component.

## Conclusion

In conclusion, TRPM7 contributes to endothelial dysfunction in primary diabetic-foot VECs in vitro and is associated with altered VEGFA-associated AKT/ERK signaling markers. These findings identify a biologically plausible TRPM7-related regulatory framework.

## Supporting information

S1 Fig Images of expression of VEGFA, p-ERK, and p-AKT detected by Western blot. (TIF)

S2 Fig Graphical Abstract. (TIF)

S1 Table Clinical characteristics of human tissue donors. (DOCX)
