# Supplementary material for: TRPM7 modulates endothelial cell functions and VEGFA-associated signaling in diabetic foot
Source: PLoS One. 2026 Oct 28;21(10):e0359834. doi: 10.1371/journal.pone.0359834 (PMC13716219; doi:10.1371/journal.pone.0359834)
Supplement: Supplementary file 1 [file pone.0359834.s003.docx]

S1 Table. Clinical characteristics of human tissue donors.

| Characteristics | Healthy Controls (n = 6) | Diabetic Foot Patients (n = 6) |
| --- | --- | --- |
| Age (years) | 55.2 ± 6.1 | 58.4 ± 5.3 |
| Sex (Male/Female) | 4月2日 | 4/2 |
| HbA1c (%) | 5.4 ± 0.3 | 8.9 ± 1.2 |
| Duration of Diabetes (years) | N/A | 12.5 ± 4.2 |
| Smoking History (Yes/No) | 2/4 | 3/3 |
| Medications |  |  |
| Insulin therapy (n) | N/A | 4 |
| Oral hypoglycemic agents (n) | N/A | 6 |
| Anti-hypertensive drugs (n) | 1 | 4 |
| Ulcer Classification (Wagner Grade) | N/A | Grade 3 (n=4), Grade 4 (n=2) |
| Severity of Infection | N/A | Moderate (n=3), Severe (n=3) |
